# Association between the *CYP2B6* polymorphisms and nonnucleoside reverse transcriptase inhibitors drug-induced liver injury: a systematic review and meta-analysis

**DOI:** 10.1038/s41598-024-79965-0

**Published:** 2024-11-27

**Authors:** Noppadol Chanhom, Janjira Sonjan, Jarupat Inchai, Wanvisa Udomsinprasert, Usa Chaikledkaew, Supharat Suvichapanich, Surakameth Mahasirimongkol, Jiraphun Jittikoon

**Affiliations:** 1 Department of Biochemistry, Faculty of Pharmacy, Mahidol University, Bangkok, 10400 Thailand; 2 Social Administrative Pharmacy Division, Department of Pharmacy, Faculty of Pharmacy, Mahidol University, Bangkok, 10400 Thailand; 3 Medical Life Sciences Institute, Department of Medical Sciences, Ministry of Public Health, Nonthaburi, 11000 Thailand

**Keywords:** Drug-induced liver injury, *CYP2B6*, Human immunodeficiency virus, Hepatotoxicity, Systematic review, Meta-analysis, Adverse effect, Genetic polymorphisms, Clinical genetics, Predictive markers, Predictive markers, Genetics research

## Abstract

Nevirapine (NVP) and Efavirenz (EFV) can cause antiretroviral drug-induced liver injury (ARVDILI). The objectives of this study were to summarize and analyze existing data on pharmacogenomics associated with nonnucleoside reverse transcriptase inhibitors drug-induced liver injury using systematic review and meta-analysis. This study systematically searched the relevant studies regarding pharmacogenes related to ARVDILI from online databases. Genes-encoding proteins were further analyzed using the STRING program to determine the protein-protein interactions (PPI). *CYP2B6* polymorphisms were further meta-analyzed. Seventeen genes have been shown to be significantly associated with ARVDILI. Illustration from STRING analysis, CYP2B6, CYP1A1, and CYP2D6 enzymes have been recognized as central proteins linked to all other analyzed proteins. Meta-analysis illustrated that CYP2B6 *1/*6 (OR = 1.83; 95% CI: 1.15–2.90; *P* = 0.01), *6/*6 (OR = 2.48; 95% CI: 1.28–4.79; *P* = 0.007), and *1/*6 plus *6/*6 (OR = 1.94; 95% CI: 1.24–3.01; *P* = 0.003) were associated with risks of EFV-induced liver injury. Moreover, CYP2B6 *1/*6 (OR = 0.44; 95% CI: 0.22–0.91; *P* = 0.03) and a group combining individuals with either *1/*6 or *6/*6 (OR = 0.42; 95% CI: 0.21–0.84; *P* = 0.01) were associated with reduced risks of NVP-induced liver injury. This meta-analysis revealed an association between *CYP2B6* genetic polymorphism and susceptibility to ARVDILI.

**Supplementary Information:**

The online version contains supplementary material available at 10.1038/s41598-024-79965-0.

## Introduction

Since its discovery in 1996, nonnucleoside reverse transcriptase inhibitors (NNRTIs) have been used in combination with other antiretroviral (ARV) medications to help reduce overall morbidity and mortality associated with human immunodeficiency virus infection^1^. Despite these beneficial features of NNRTIs, which significantly improves the prognosis and quality of life of people living with Human Immunodeficiency Virus (PLHIV), some PLHIV with ARV develop severe adverse drug reactions (ADRs), especially drug-induced liver injury (DILI). The reported incidence of ARVDILI can range from 8 to 23% of PLHIV; nevertheless, up to 30% of these PLHIV require a change of regimen or treatment discontinuation, which subsequently causes prolonged hospitalization, in which severe cases will be fatal^2–5^, If not detected and treated promptly, it may potentially cause severe hepatitis and even death in some PLHIV^6,7^.

Nevirapine (NVP) is the NNRTI used in countless PLHIV due to its proven efficacy, advantageous metabolic profile, cost-effectiveness, and particularly its significance in strategies aimed at simplifying combination antiretroviral therapy (cART)^8–10^. Unfortunately, approximately 6–17% of NVP recipients experienced ARVDILI, with up to 12% developing severe ARVDILI^11–14^. In terms of its biological metabolism, cytochrome P450 primarily facilitates the biological metabolism of NVP, resulting in the formation of four mono-oxygenated metabolites. 12-OH-NVP, a byproduct of CYP, has reportedly been associated with the development of rash, which may play a role in hepatic toxicity^15^. In addition to NVP, Efavirenz (EFV), one of the NNRTIs, is used in numerous successful first-line HIV regimens. However, efficacy, tolerability, and availability made it an exception for certain PLHIV^16^. Unfortunately, EFV administration was associated with ARVDILI. Drug accumulation in the liver was considered the perpetrator^17^. To increase their safety, it is essential to fill the gap in knowledge about NNRTIs’ effects on the liver and their role in DILI development^18^. Interestingly, genetics has been hypothesized as one of the critical contributors to ARVDILI pathogenesis in several populations^19^.

According to several findings from previous studies as a putative mediator for liver disease in PLHIV, *CYP2B6* is the most promising of numerous intriguing genes associated with ARVDILI. It is responsible for several drugs metabolism and synthesizing cholesterol, steroids, and other lipids^20^. Interestingly, CYP2B6 enzymes are also the primary metabolizers of nevirapine and efavirenz. In all investigated populations, CYP2B6*6 is the most common variant allele^20^. CYP2B6*6 haplotype is characterized by the existence of two variants, G516T (rs3745274) and A785G (rs2279343), with or without promoter variants^21^. This allele has been associated with a 50–75% decrease in CYP2B6 enzyme levels but not with the protein’s active or substrate recognition sites^22–24^. In that context, a decrease in protein level might help explain why CYP2B6*6 was associated with an increased risk of ARVDILI from efavirenz^25^ and nevirapine^26^. In contrast to the above findings, other previous studies^13,27,28^ unveiled no correlation between CYP2B6*6 polymorphisms and susceptibility to ARVDILI due to efavirenz as well as nevirapine. To clarify the above associations, a meta-analysis, a valuable tool for deriving meaningful conclusions from data and for resolving inconsistencies found in research, is needed.

Accordingly, the objectives of this study were to summarize and analyze existing data on pharmacogenomics associated with nonnucleoside reverse transcriptase inhibitors drug-induced liver injury using systematic review and meta-analysis approaches along with network analysis to gain insight into molecular interactions of these pharmacogenes and their genetic polymorphisms associations with ARVDILI.

## Results

### A systematic review of genetic polymorphisms associated with ARVDILI

Eight hundred forty-one publications were collected from PubMed, Scopus, and Cochrane databases using the search terms specified in methodologies. After data refinement using exclusion criteria, 165 duplicated publications were removed. In addition, 377 publications were excluded due to unrelated topics, 247 were not primary sources of data, 2 were studies in vitro and in silico models, and one was published in a language other than English. In the end, 50 different papers were considered for inclusion in this study.

However, 39 publications reported on associations of genetic polymorphisms with ARVDILI. The top-fifth of the highest number of reports were 11 for *UGT1A1*, 11 for *CYP2B6*, four reports of *ABCB1*, two for *HLA-Cw*, and two for *HLA-DRB1* (Figure S1). The protein-protein interaction (PPI) analysis of collected ARVDILI-associated genes depicted interactions among several genes. In the pathway collected by the Kyoto Encyclopedia of Genes and Genomes (KEGG), alternative findings from a PPI network showed that the network is functionally enriched in the metabolism of xenobiotics^29^. The results illustrated that the ARVDILI was primarily involved in xenobiotic metabolism. Interestingly, the STRING database indicates that CYP2D6, CYP1A1, and CYP2B6 seem to be the center of the interaction among those proteins (Figure S2). In PPI analysis, CYP2B6 was selected as a candidate gene due to its frequent reports and centrality. From this, *CYP2B6* genetic polymorphisms were further analyzed using the Cochran-Mantel-Haenszel analysis approach.

### Meta-analysis of *CYP2B6* associated with efavirenz-induced liver injury

#### Study selection and characteristics

**Fig. 1:**
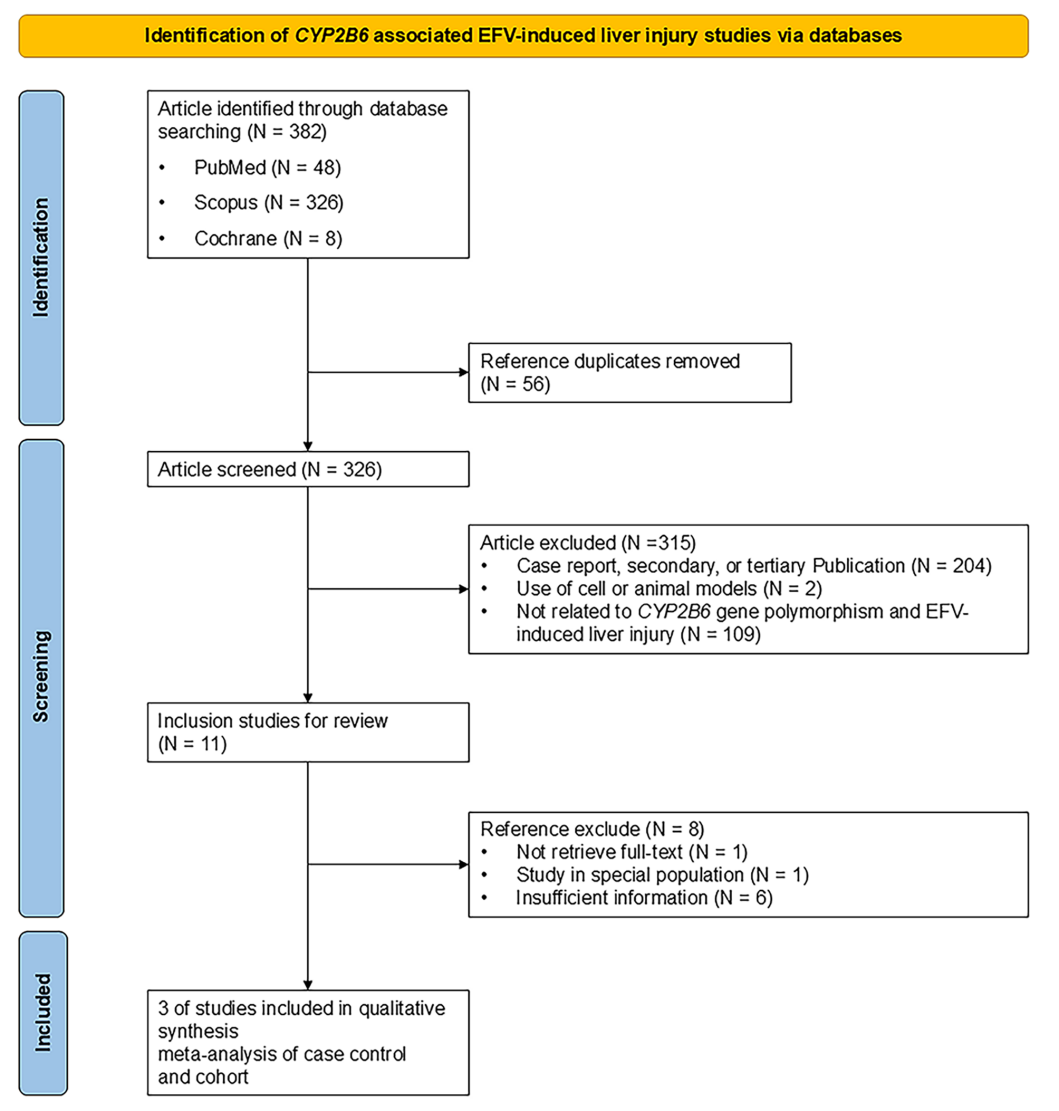
Flow diagram of this meta-analysis of CYP2B6 association with EFV-induced liver injury. A total of 350 publications were retrieved from databases up to 10 October 2024. Three studies were selected as eligible publications after the screening and quality assessment process.

The selection process is registered in PROSPERO (ID: CRD42024593948) and illustrated in Fig. 1. Initially, 350 publications were included in this study. Publications were initially included for publication appraisal. Following the Newcastle-Ottawa scale for case-control and cohort study, three publications with 106 cases of ARVDILI from a total of 795 PLHIV were selected. The publication years ranged from 2011 to 2012, as described in Table 1. All the studies were conducted on the African population. There were two cohort studies and one case-control study.

**Table 1:** Characteristics of reference studies.

References	Study design	Country	Ethnicity	Control Source	DILI definition	ARVDILI (case)	Non-ARVDILI (control)	Mean age of the case	Mean age of control	Sex in case (male/female)	Sex in control (male/female)
Publications in the Efavirenz group											
Mugusi et al. (25)	Cohort	Tanzania	African	HB	CIOMS	28	321	41.81	39.78	21/16	182/254
Yimer et al. (28)	Cohort	Ethiopia	African	HB	CIOMS	37	208	NA	NA	9/32	66/154
Yimer et al. (27)	Case-control	Ethiopia	African	HB	CIOMS	41	160	NA	NA	26/39	140/148
Publication in Nevirapine group											
Carr et al. (26)	Case-control	Malawi	African	HB	ALT > 40 IU/L with jaundice	30	428	37.4	36.2	78/131	205/258
	Case-control	Uganda	African	HB		29	460	37.4	36.2		
Giacomelli et al. (29)	Case-control	Italy	Caucasian	NA	RUCAM	8	354	39.6	38.5	2/6	227/127

CIOMS, Council for International Organizations of Medical Science; HB, hospital-based; NA, not available; RUCAM, Roussel Uclaf Causality Assessment Methods.

#### Meta-analysis results

**Fig. 2:**
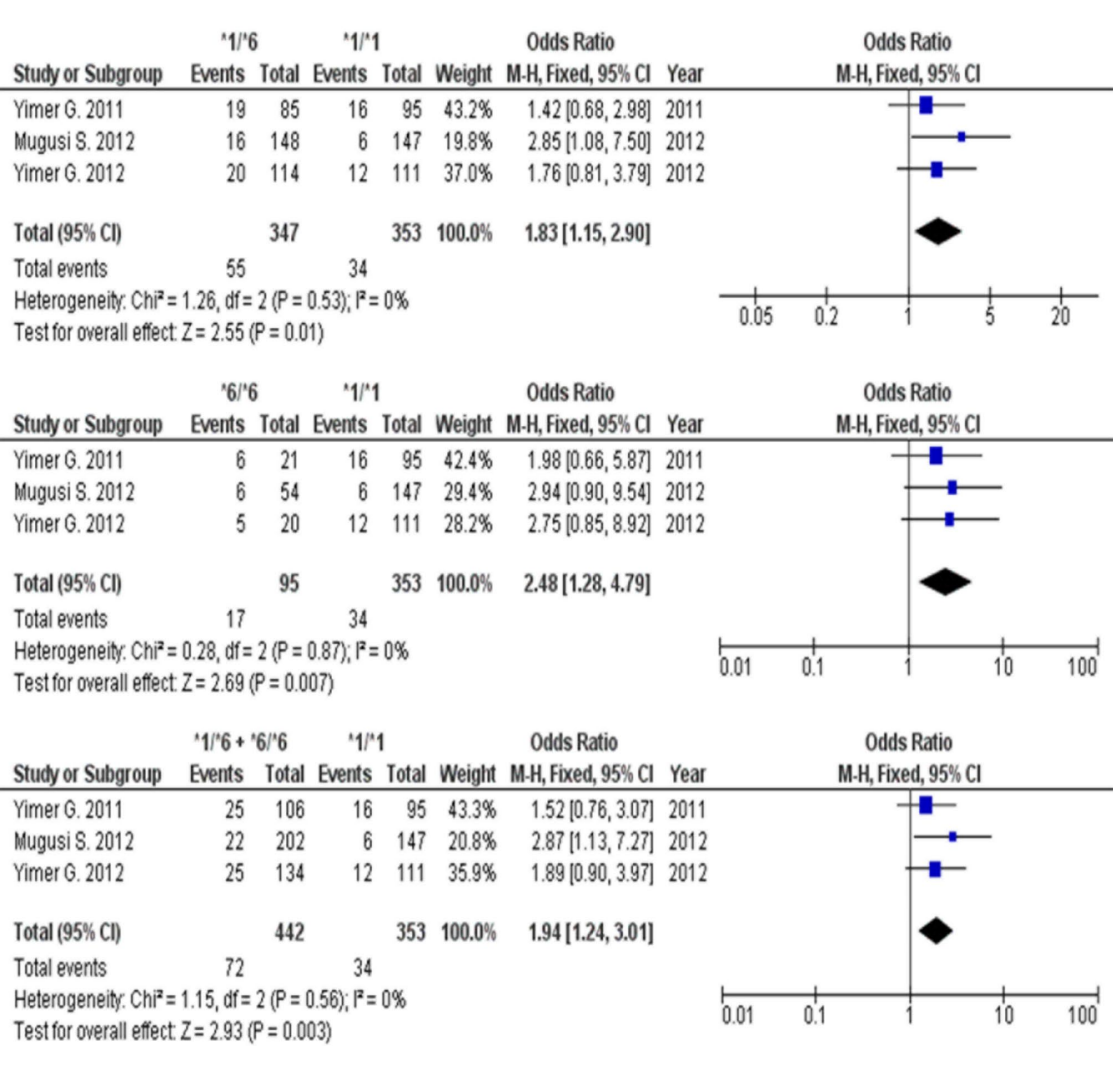
Forest plot comparison between *CYP2B6* variance and wild-type and the susceptibility of EFV-induced liver injury from 3 publications. CI, confidential interval.

The pooled effects of *CYP2B6* polymorphisms on EFV-induced liver injury occurrences in PLHIV are summarized in Table 2 and illustrated in Fig. 2. Compared with normal metabolizers, CYP2B6 *6 carriers were significantly more susceptible to EFV-induced liver injury, with an odds ratio (OR) and 95% confidence interval (CI) of 1.83 (1.15–2.90), 2.48 (1.28–4.79), and 1.94 (1.24–3.01) for heterozygous, homozygous, and combination of both alleles, respectively, as well as *P* value less than 0.05 (Table 2).

**Table 2:** Summary of meta-analyses between *CYP2B6* genotypes and the risk of ARVDILI.

CYP2B6 genotype compared with CYP2B6 *1/*1	Test of heterogeneity		Effects model	Mutant genotype count		Normal metabolizer genotype counts		Total	Number of included studies	OR	95% CI	*P*-value
	I^2^(%)	*P*-value		case	control	case	control					
Efavirenz-induced liver injury												
*1/*6	0	0.53	Fixed	55	292	34	319	700	3	1.83	1.15–2.90	0.01
*6/*6	0	0.87	Fixed	17	78	34	319	448	3	2.48	1.28–4.79	0.007
*1/*6 + *6/*6	0	0.56	Fixed	72	370	34	319	795	3	1.94	1.24–3.01	0.003
Nevirapine-induced liver injury												
*1/*6	0	0.97	Fixed	15	383	19	297	714	2	0.44	0.22–0.91	0.03
*6/*6	0	0.85	Fixed	4	102	19	297	533	2	0.45	0.15–1.32	0.14
*1/*6 + *6/*6	0	0.89	Fixed	19	485	19	297	820	2	0.42	0.21–0.84	0.01

ARVDILI, antiretroviral drug-induced liver injury; CI, confidence interval; OR, odds ratio.

#### Sensitivity, heterogeneity, and publication bias analyses

To determine the impact that each study had on the total estimate, a sensitivity analysis was performed in which a single study was excluded at a time. The findings of the study indicated that the sensitivity test for the meta-analysis of *CYP2B6* genotypes was successfully completed. Heterogeneity had not been noticed. A funnel plot was utilized in order to analyze publication bias. The condensed plot that is provided at the top of the 95% confidence interval triangle reveals publication bias for studies that had a large number of PLHIV participants, as shown by the funnel plots. But the plots were symmetrical between the sides of the triangle, which showed that there was no publication bias observed for either negative or positive results (Figure S3).

### Meta-analysis of *CYP2B6* associated with nevirapine-induced liver injury

#### Study selection and characteristics

**Fig. 3:**
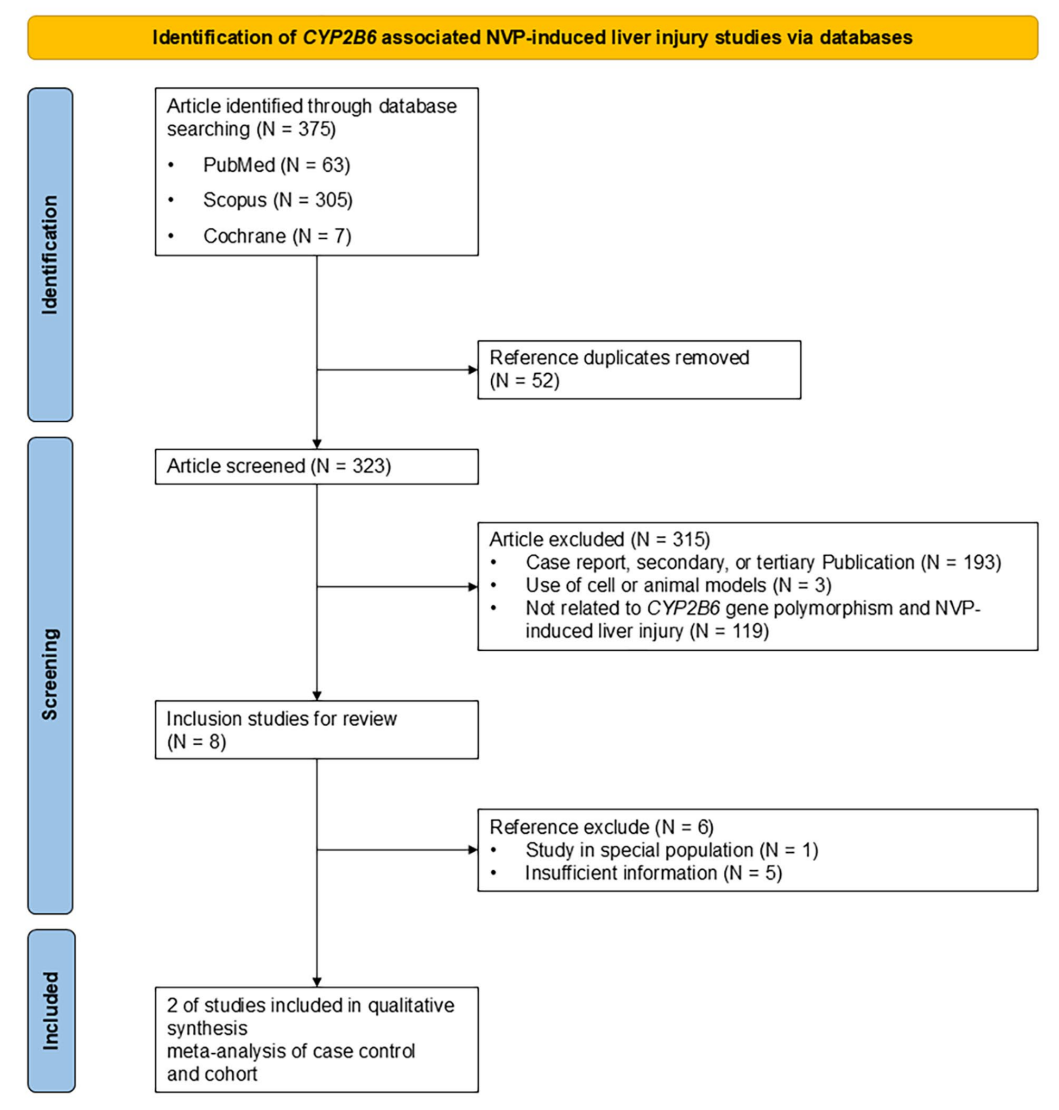
Flow diagram of this meta-analysis of *CYP2B6* association with NVP-induced liver injury. A total of 347 publications were retrieved from databases up to 10 October 2024. Two studies were selected as eligible publications after the screening and quality assessment process.

The selection process is registered in PROSPERO (ID: CRD42024593948) and demonstrated in Fig. 3. To begin, 356 publications were included for selection. Then, due to duplication, 49 publications were removed. Following that, 307 publications were screened, with 305 excluded from the analysis; most studies fulfilled the exclusion criteria, while some lacked eligible data for analysis. Finally, two publications were included for the appraisal (Supplementary Table S1), including 38 ARVDILI and 782 non-ARVDILI PLHIV (Table 1).

#### Meta-analysis results

**Fig. 4:**
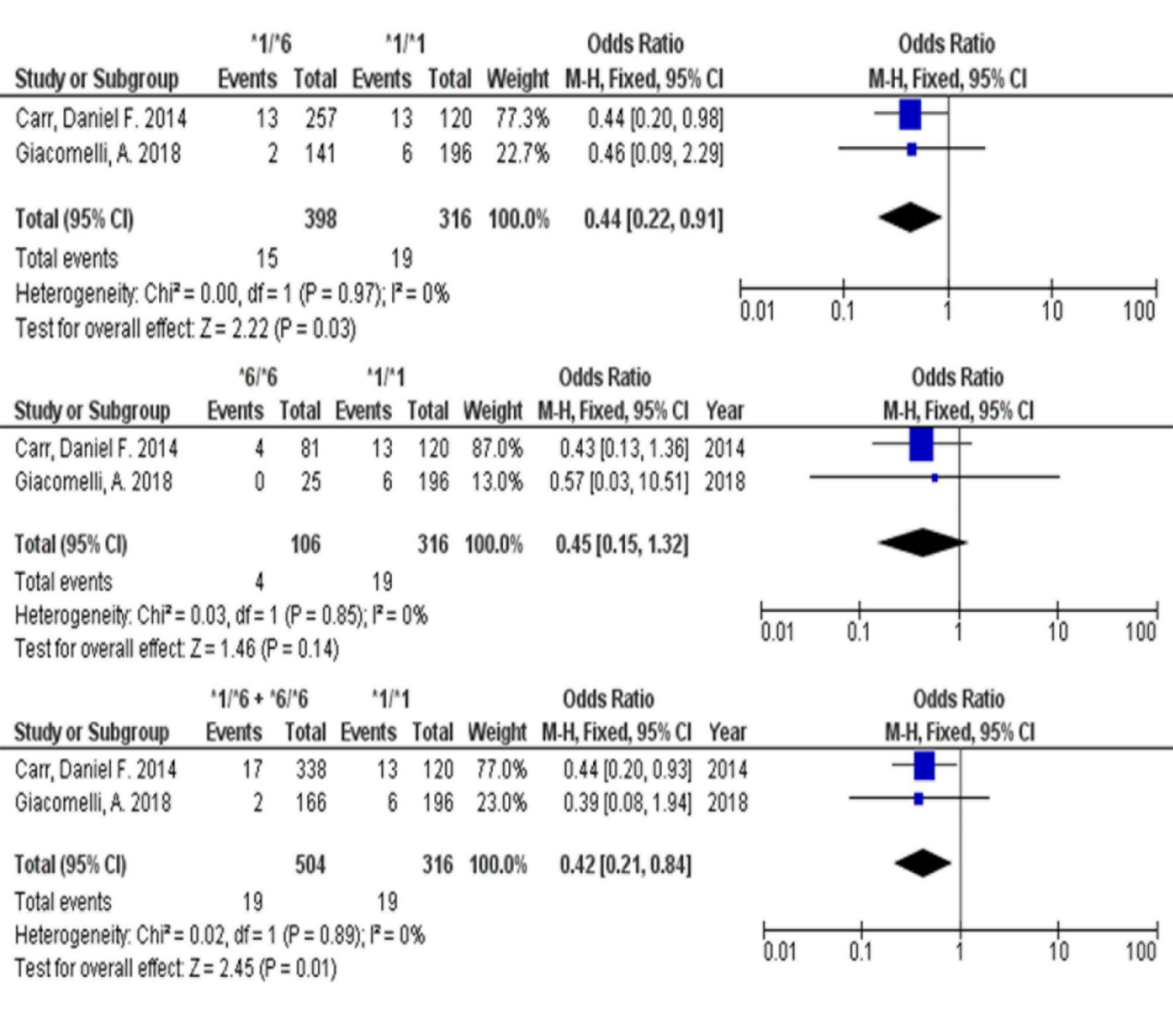
Forest plot comparison between *CYP2B6* variance and wild-type and the susceptibility of NVP-induced liver injury from 2 publications. CI, confidential interval.

The association of *CYP2B6* with NVP-induced liver injury sensitivity in PLHIV is detailed in Table 2. Meta-analysis showed that CYP2B6 *1/*6 allele and combination of *1/*6 + *6/*6 alleles were significantly associated with a reduction in susceptibility to NVP-induced liver injury, compared with normal metabolizer (Fig. 4), with pooled OR and 95% CI of 0.44 (0.22–0.91) and 0.42 (0.21–0.84), respectively, as well as *P* value less than 0.05 (Table 2). However, there was no significant association between *CYP2B6* homozygous *6 with ARVDILI risk (Fig. 4).

#### Sensitivity, heterogeneity, and publication bias analyses

According to the results of the sensitivity analysis, the substantial relationships between the CYP2B6 *1/*6 allele and a combination of *1/*6 + *6/*6 alleles with a lower risk of NVP-induced liver injury were not attributable to chance. Heterogeneity had not been noticed. An investigation on publication bias was carried out utilizing a funnel plot, which revealed that publication bias was not discovered for either a negative or positive result (Figure S4).

## Discussion

Recently, genetic polymorphisms associated with ARVDILI have been studied, increasing the possibility of utilizing them as biomarkers to predict the chance of developing ARVDILI^28^. Consistent with this, our systematic review showed numerous reports on the relationship between genetic polymorphisms and ARVDILI in several populations. The fifth highest frequency of reported gene-associated ARVDILI was *UGT1A1*, *CYP2B6*, *ABCB1*, HLA-Cw*04, and *HLA-DRB1*, respectively.

Additionally, the PPI analysis illustrated the functional enrichment of the protein in the network involved in the metabolism of xenobiotics according to the Kyoto Encyclopedia of Genes and Genomes (KEGG). The results suggested that the ARVDILI was mainly affected by the function of xenobiotic metabolisms. In addition, PPI network analysis of these gene-encoded proteins demonstrated links between those proteins with CYP1A1 and CYP2B6 as central to their interactions. The result demonstrated the association of CYP1A1 and CYP2B6 proteins with other proteins in the network. Due to the centrality in the PPI network and reports frequency, the CYP2B6 protein has been hypothesized to be the crucial molecule.

Consequently, based on this premise, we conducted meta-analyses to verify the relationship between *CYP2B6* polymorphisms and their association with ARVDILI susceptibility. Our findings demonstrated that a genotype known as CYP2B6*6, which is related with a decreased ability to metabolize drugs, was linked to an increased risk of ARVDILI in PLHIV patients who were using EFV. Conversely, the mentioned genotype is also associated with a reduced risk in nevirapine-administered PLHIV.

CYP2B6 is the drug-metabolizing enzyme involved in EFV and NVP metabolisms, especially EFV. Plentiful evidence obtained from hepatocyte experiments uncovered that EFV compromised cell viability and altered mitochondrial function by diminishing mitochondrial membrane potential, decreasing mitochondrial O_2_ consumption, and increasing ROS production^30–33^. In addition to EFV, 8-hydroxyefavirenz (8-OH-EFV), the primary metabolite of EFV, generally has the capability to induce cell death and ROS production in hepatocytes^34^. Therefore, the EFV and its primary metabolite are the reactive forms that can cause ARVDILI in PLHIV. In addition, according to a previous study, the CYP2B6 enzyme metabolizes EFV into 8-OH-EFV, then 8,14-diOHEFV, respectively^35^. Therefore, it is unsurprising that the poor metabolizer form of the CYP2B6 enzyme, such as *6, has been linked to ARVDILI in several populations.

On the contrary, in NVP’s circumstance, the meta-analysis results showed that *CYP2B6* *1/*6 and a combination of *1/*6 and *6/*6 were dominantly found in the controls group, thus indicating that the poor metabolizer form of CYP2B6 may reduce the risk of ARVDILI. In contrast to this result, the LiverTox database and a previous study by Ciccacci et al. both depicted that the CYP2B6 inducer produced a toxic intermediate and subsequently caused DILI in PLHIV^36,37^. This contradictory result may be explained by the fact that NVP has several metabolizing enzymes and numerous metabolites, resulting in the production of 2-, 3-, 8-, and 12-hydroxynevirapine (-OH-NVP) and 4-carboxynevirapine. Unlike EFV, the active metabolite produced by CYP2B6, 8-OH-NVP, is not the most abundant metabolite. Moreover, the major metabolite of NVP produced by CYP3A4, 12-OH-NVP^38^, has been implicated in hepatotoxicity and skin rash caused by NVP^15,39,40^. Therefore, to draw a clear explanation of the association between *CYP2B6* polymorphisms and hepatotoxicity in NVP-administered PLHIV, the genotypic information of *CYP3A4* is required. This discrepancies between EFV and NVP results may cause different interpretation of *CYP2B6* genotyping results between two medication users. While poor metabolizer of CYP2B6 may cause DILI in EFV using PLHIV and should decide the regimen alteration, NVP using PLHIV may continue without any interaction. However, due to the limited number of publications and sample size, another study on this topic is encouraged to identify the exact reason for conflicting results.

The CYP2B6 enzyme is not the exclusive metabolizer of EFV and NVP^41^. UGTs, CYP3A4, CYP3A5, CYP1A2, and CYP2A6 may have minimal contributory roles in the xenometabolic phase of EFV^22,42–44^. CYP3A4, CYP3A5, CYP2C9, and CYP2D6 are also involved in the metabolism of NVP^45–47^. Consequently, the influence of several genes on ARVDILI susceptibility seems inescapable. Chanhom et al. observed the effects of various genes in ATDILI within the Thai population^48^. This finding also indicates the potential for various gene association studies in ARVDILI. Nevertheless, further studies are needed to investigate the specific interactions between these genes and ARVDILI susceptibility, especially in diverse populations. Understanding the role of genetic variations in drug metabolism can help improve personalized medicine and reduce the risk of adverse reactions. Overall, the complex interplay between genes and DILI underscores the importance of pharmacogenomics in optimizing patient care.

Noteworthy, some inherent limitations need to be considered when evaluating the presented findings. First, we included publications published only in English; thus, language bias was unavoidable. Accordingly, we lost reports published in other languages, possibly leading to selection bias. Moreover, we performed a PPI analysis to investigate the potential of the protein/gene for further meta-analysis. However, the PPI function is quite restricted and cannot be confirmed by clinical outcomes. The authors encouraged further bioinformatic study to investigate the interplay among genes, proteins, and clinical outcomes in ARVDILI. Moreover, one of the possible confounders was DILI definition due to the change of the definition in 2016^49^ which may affect the DILI interpretation discrepancies between studies. The author encouraged further observational studies or trials to comply with the updated RUCAM criteria for DILI causality assessment^49^. Along with this, some publications were excluded due to our rigid selection protocol, which might be the possible reason for sparse publications and sample sizes and could cause inconsistent results, especially in NVP-induced liver injury. Furthermore, the limited number of publications and sample size in this meta-analysis adversely affects the generalizability of its conclusions. The meta-analysis of the link between *CYP2B6* genotypes and EFV-induced liver injury was only derived from the African population, as well as NVP-induced liver injury were obtained from only African and Caucasian populations. Consequently, referencing this may produce divergent outcomes in other populations with varying polymorphism frequencies. Contrastingly, this meta-analysis highlights the research deficit in different populations, and a rigid selection protocol might construct an exceptional foundation for future studies. For this reason, we might mitigate the effect of the confounding factors, which provide us with more precise analysis results. Notably, this is the first systematic review and meta-analysis on the association between *CYP2B6* polymorphisms and ARVDILI in PLHIV.

In conclusion, our systematic review illustrated that *UGT1A1*, *CYP2B6*, and *ABCB1* were the most frequently studied genes. This meta-analysis illustrated that CYP2B6 *6 and *1 was significantly associated with EFV-induced liver injury and NVP-induced liver injury in PLHIV, respectively. Additionally, the results of this study may use as a fundamental knowledge to develop decision support guideline for drug selection and personalized medicine for PLHIV in the near future. Further observational studies with larger sample size, well-characterized subjects, and various ethnicities are warranted to gain a precise conclusion and develop those polymorphisms as genetic markers for predicting PLHIV with a high ARVDILI risk induced by EFV and NVP.

## Materials and methods

### Gene associated with ARVDILI exploration

Scopus and PubMed were combed using the following search terminology. ((“ARV” OR “Antiretroviral treatment” OR “antiretroviral agent*” OR “Antiretroviral drug*"OR “Anti-HIV Agent*” OR “HIV therapy” OR “HIV treatment” OR “HAART” OR “NNRTI*” OR “nonnucleoside reverse transcriptase inhibitors”) AND (“genetic polymorphism*” OR “polymorphism*” OR “Pharmacogenetic*” OR “Immunogenetic*” OR “Pharmacogenomic*” OR “genome wide association study” OR “variant allele*” OR “genetic predisposition” OR “Gene Variant*”)) AND (“ARV-associated hepatotoxicity” OR “drug-induced hepatotoxic” OR “Drug-induced hepatotoxicity” OR “Hepatotoxic” OR “Hepatotoxicity” OR “Liver toxicity” OR “Liver toxic” OR “drug-induced liver injury” OR “DILI” OR “liver injury” OR “Drug induced hepatitis” OR “Drug-induced hepatitis” OR “Hepatitis” OR “hypersensitivity reaction” OR “hypersensitivity*” OR “hyperbilirubinemia”). From inception to 1 August 2023, original research articles on the association between genetic polymorphisms and ARVDILI were gathered. The studies with negative results were exclusively excluded from the analysis. Frequencies of association reports of each gene-ATDILI association were reported. Proteins associated with ARVs were analyzed using STRING online software version 11.5 (https://string-db.org/)^50^. The STRING integrated and classified protein/gene associations by benchmarking them in experimental and predicted physical/theoretical protein-protein interactions data. By the criteria of the STRING standard, the interaction network was deemed to have a confidence score of more than 0.70, and all interaction sources were selected, including text-mining, experiment, databases, co-expression, neighborhood, gene fusion, and co-occurrence. Each protein was represented as a node in the protein-protein interaction (PPI) network, and an interaction between two proteins in the network was denoted by an edge. These interactions might either be physical or theoretical.

### Meta-analysis of *CYP2B6* associated with efavirenz/nevirapine-induced liver injury

#### Search strategies of the literature for meta-analysis

On 10 October 2024, the original publication search was performed using electronic databases of PubMed, Scopus, and Web of Science. The PICO strategy was used throughout the search process^51^. Following is the definition of the PICO: Population (P): PLHIV who receive efavirenz or nevirapine medication, Intervention (I): *CYP2B6* genotype associated with an increased risk of ARVDILI in PLHIV, Comparator (C): *CYP2B6* normal metabolizer, and Outcomes (O): hepatotoxicity. The search techniques were developed by combining several search phrases with various Boolean operators^52^.

#### The process of selecting studies for meta-analyses

The inclusion criteria were as follows: (i) the study subjects were PLHIV receiving EFV or NVP; (ii) the study investigated the association between *CYP2B6* polymorphisms and ARVDILI susceptibility; and (iii) the study was a randomized controlled trial, cohort, or case-control design. The exclusion criteria were as follows: (i) the study was either a case report secondary or tertiary publication such as a review, systematic review, and meta-analysis, (ii) the study was conducted in an animal model, in vitro, or in silico, iv) full article cannot be accessed, (iii) the study was not published in English, (iv) the study did not observe the liver function test, (v) the study included confounding factors such as viral hepatitis infection, alcoholism, or concomitant hepatotoxic medication, (vi) the study included vulnerable subjects such as children, pregnant/breastfeeding women, or elderly, (vii) the study is unrelated to the association between at *CYP2B6* polymorphisms and ARVDILI susceptibility or does not have sufficient data for analysis.

#### Assessment of selected study quality

The Newcastle-Ottawa scale was utilized by two reviewers, JS and JI, in order to conduct independent evaluations of the articles’ overall quality^53^. The issue that existed between the two reviewers was finally settled by conversation and agreement with the third reviewer (NC). The scores on the Newcastle-Ottawa scale range from 0 to 9, with 0 being the lowest and 9 being the highest. If the publication’s overall evaluation score was higher than 6, then it was determined that the publication successfully passed the evaluation (Supplementary Table 1). The studies were grouped according to the medication involved in the study, i.e., either the NVP group or the EFV group.

#### Data extraction for meta-analysis

The information from each of the research that were considered to be relevant was compiled into a single sheet of data. The following information was taken from the article: first author, publication year, study design, ethnicity, sample size, gender, age, BMI, observed medication, DILI definition and the number of ARVDILI/non-ARVDILI for each *CYP2B6* genotype along with odds ratios and 95% confidence intervals. In the event where two of the reviewers, JS and JI, could not come to an agreement regarding the findings of the data extraction, the issue was settled through discussion and by reaching a consensus with the third reviewer (NC).

### Statistical analysis

Meta-analysis of *CYP2B6* gene effect sizes was conducted using Computer program Review Manager (RevMan^®^). Version 5.3. Copenhagen: The Nordic Cochrane Centre, the Cochrane Collaboration, 2014. In accordance with low heterogeneity among studies and similarity of population and study design, The Mantel-Haenszel fixed-effects model was used to calculate pooled OR, 95% CI, and P-value^54^. In order to be declared statistically significant, the P-value has to be lower than 0.05. The Chi-square test or an I^2^ value of more than 50% were utilized to make an approximation of the heterogeneity that existed among the studies^55^. The results of the individual research and the synthesis were plotted out in the forest plot so that they could be viewed visually. A funnel plot was then used to establish whether or not there was any publishing bias.

## Electronic supplementary material

Below is the link to the electronic supplementary material.

Supplementary Material 1

## Data Availability

Data is provided within the manuscript or supplementary information files.

## Abbreviations

ARVDILI: Antiretroviral drug-induced liver injury
cART: Combination antiretroviral therapy
CI: Confidence interval
DILI: Drug-induced liver injury
EFV: Efavirenz
NVP: Nevirapine
OR: Odds ratio
PLHIV: People living with HIV
PPI: Protein-protein interaction
