# Supplementary material for: Association between the CYP2B6 polymorphisms and nonnucleoside reverse transcriptase inhibitors drug-induced liver injury: a systematic review and meta-analysis
Source: Sci Rep. 2024 Nov 27;14:29511. doi: 10.1038/s41598-024-79965-0 (PMC11603346; doi:10.1038/s41598-024-79965-0)
Supplement: Supplementary file 1 — Supplementary Material 1 [file 41598_2024_79965_MOESM1_ESM.docx]

**Supplementary Figures**


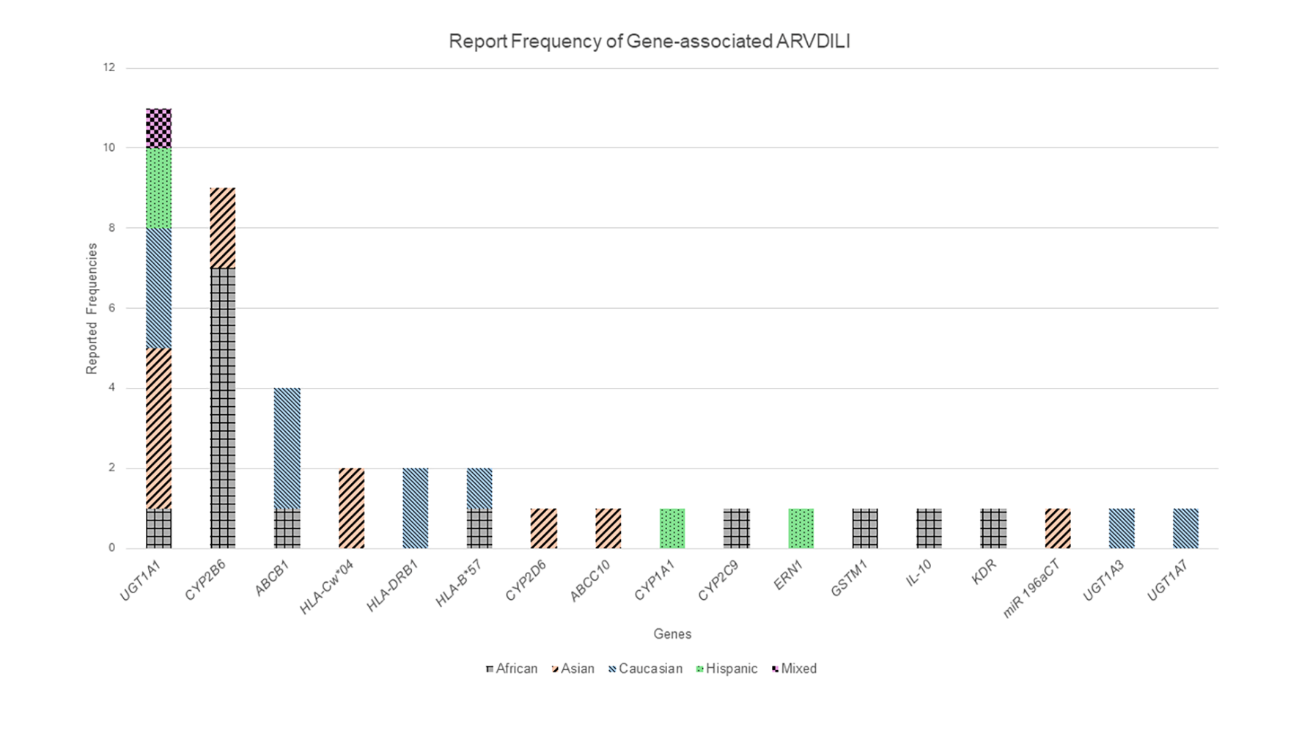


Figure S1. Report frequencies of gene-associated ARVDILI in PubMed and Scopus databases. The reports were searched up to 14 January 2022 and separated according to studied ethnicity.


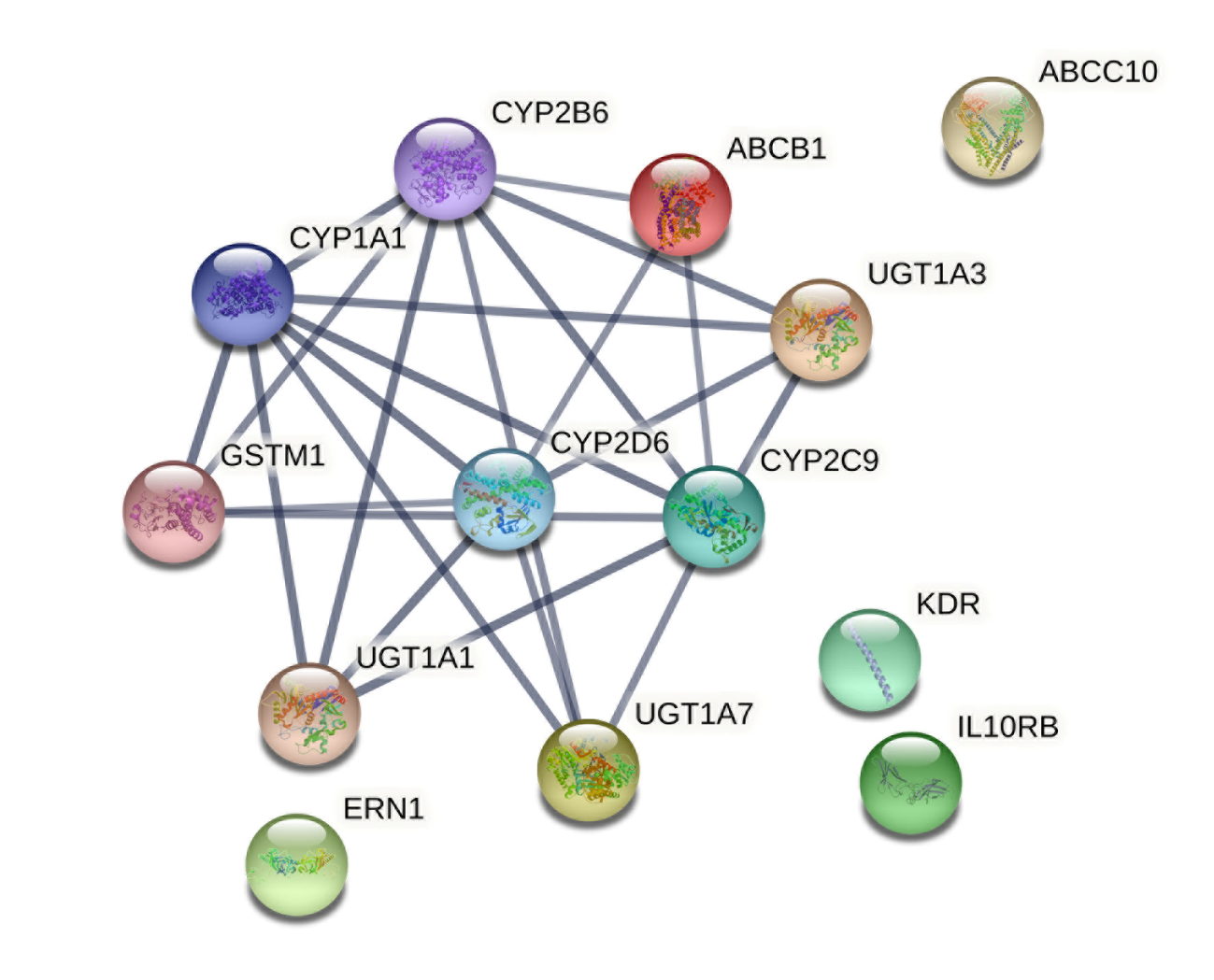


Figure S2. Protein-protein interaction network of the gene associated ARVDILI with high interaction score of higher than 0.7. Each node represents each protein in this pathway.


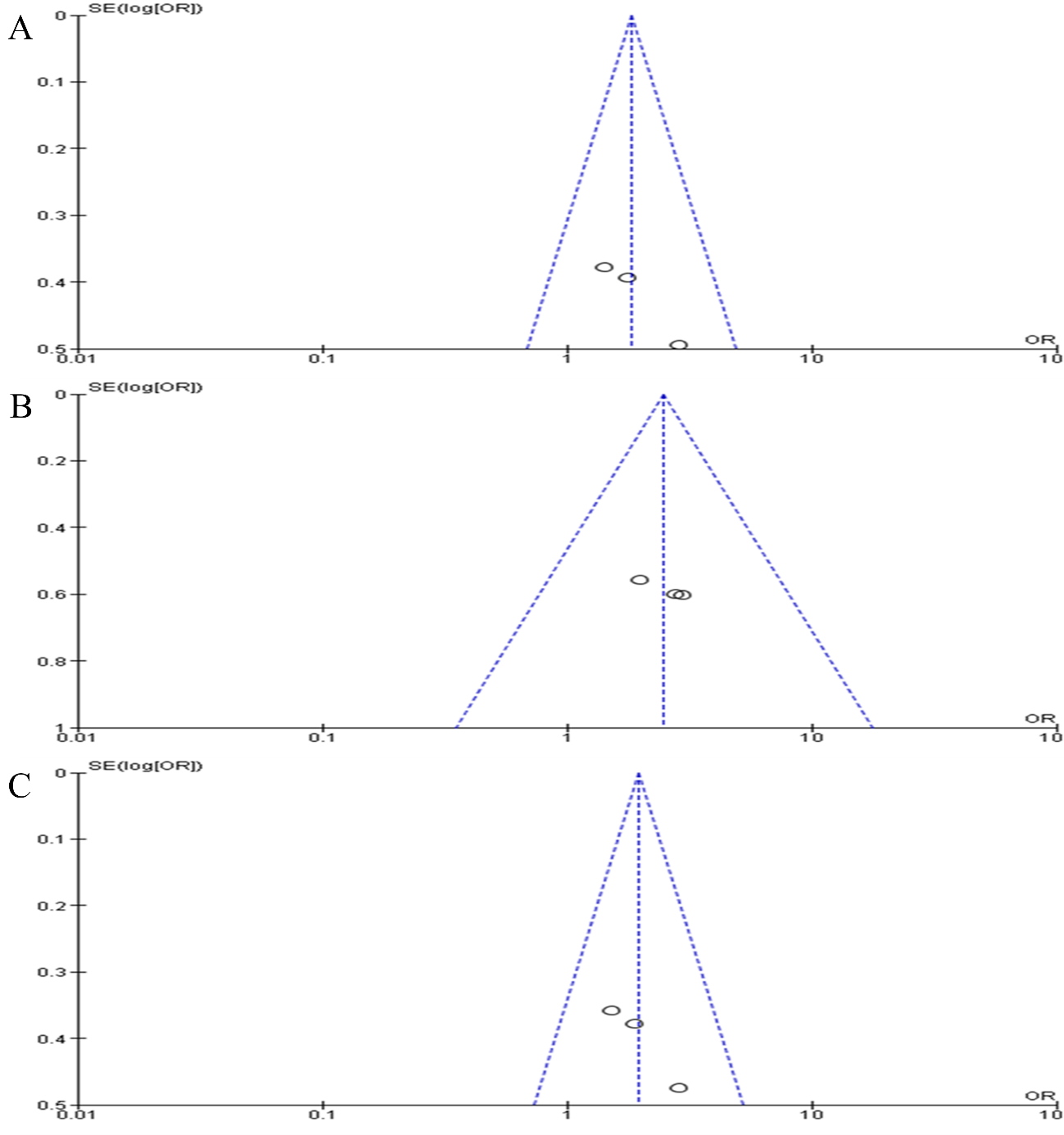


**Figure S3. Funnel plot of the comparison between *CYP2B6* variance and wild-type and the susceptibility of EFV-induced liver injury from 3 publications.** (A) Funnel plot of the comparison between *CYP2B6* *1/*1 and *1/*6. (B) Funnel plot of the comparison between *CYP2B6* *1/*1 and *6/*6. (C) Funnel plot of the comparison between *CYP2B6* *1/*1 and *1/*6 plus *6/*6


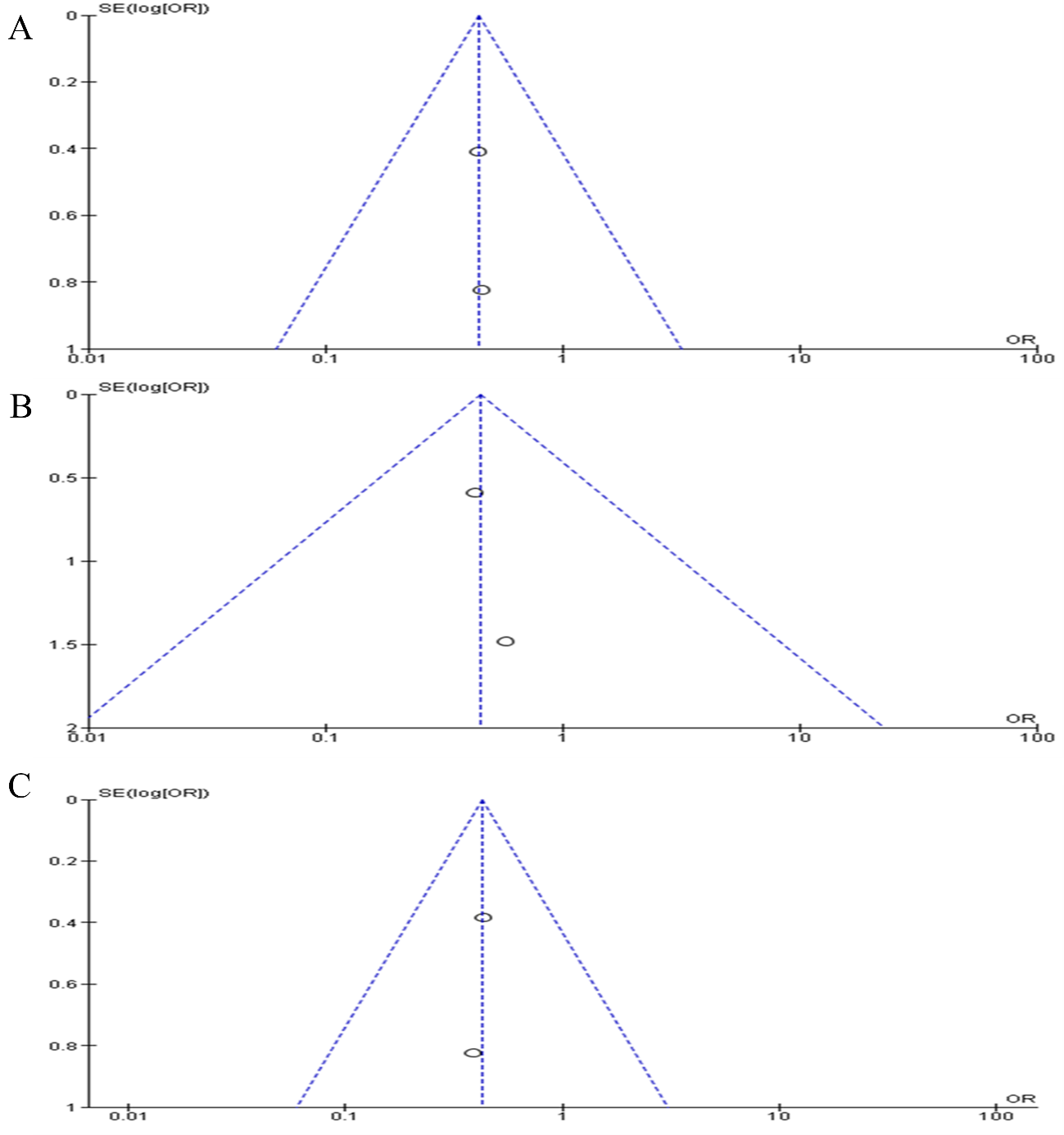


**Figure S4. Funnel plot of the comparison between *CYP2B6* variance and wild-type and the susceptibility of NVP-induced liver injury from 2 publications.** (A) Funnel plot of the comparison between *CYP2B6* *1/*1 and *1/*6. (B) Funnel plot of the comparison between *CYP2B6* *1/*1 and *6/*6. (C) Funnel plot of the comparison between *CYP2B6* *1/*1 and *1/*6 plus *6/*6

**Supplementary table**

**Table S1. Publication appraisal using The Newcastle-Ottawa Scale (NOS) for assessing the quality of case-control/Cohort study**

| No | Author | Year | Study design | Selection | | | | Comparability | Exposure/Outcome | | | Total |
| --- | --- | --- | --- | --- | --- | --- | --- | --- | --- | --- | --- | --- |
|  |  |  |  | 1 | 2 | 3 | 4 | 5 | 6 | 7 | 8 | 9 |
| 1 | Yimer et al. (24) | 2011 | Case-control | * | * | * | * | ** | * | * |  | 8 |
| 2 | Yimer et al. (23) | 2012 | Cohort | * | * | * | * | ** | * | * | * | 9 |
| 3 | Mugusi et al. (21) | 2012 | Cohort | * | * | * | * | ** | * | * | * | 9 |
| 4 | Carr  et al. (22) | 2014 | Case-control | * | * | * | * |  | * | * |  | 6 |
| 5 | Giacomelli  et al. (25) | 2018 | Case-control | * | * | * | * | ** | * | * | * | 9 |
